# The COVID-19 Pandemic and Patient Expectations About Recovery From Acute Respiratory Failure

**DOI:** 10.1001/jamanetworkopen.2024.44318

**Published:** 2024-11-08

**Authors:** Diana C. Bouhassira, Victor D. Dinglas, Emma M. Lee, Sarah Beesley, Harris Carmichael, James C. Jackson, Mustafa Mir-Kasimov, Carla M. Sevin, Somnath Bose, Valerie Goodspeed, Ramona O. Hopkins, Samuel M. Brown, Dale M. Needham, Alison E. Turnbull

**Affiliations:** 1 Pulmonary and Critical Care Medicine, Johns Hopkins University, Baltimore, Maryland; 2 Outcomes After Critical Illness and Surgery Group, Johns Hopkins University, Baltimore, Maryland; 3 University of Colorado School of Medicine, Denver; 4 Pulmonary and Critical Care Medicine, Intermountain Medical Center, Salt Lake City, Utah; 5 Pulmonary and Critical Care Medicine, University of Utah, Salt Lake City; 6 Center for Humanizing Critical Care, Intermountain Medical Center, Salt Lake City, Utah; 7 Allergy, Pulmonary, and Critical Care Medicine, Vanderbilt University Medical Center, Nashville, Tennessee; 8 Salt Lake City Veterans Administration, Salt Lake City, Utah; 9 Anesthesia, Critical Care and Pain Medicine, Beth Israel Deaconess Medical Center, Boston, Massachusetts; 10 Psychology Department, Brigham Young University, Provo, Utah; 11 Neuroscience Center , Brigham Young University, Provo, Utah

## Abstract

This cohort study investigates the association of the COVID-19 pandemic with expectations about recovery among survivors of non–COVID-19 acute respiratory failure.

## Introduction

Expectations about recovery affect quality of life after intensive care among survivors of acute respiratory failure (ARF).^1,2^ We hypothesized that increased exposure to information about ARF outcomes during the COVID-19 pandemic would be associated with lowered expectations about recovery among survivors of non–COVID-19 ARF treated during that time.

## Methods

In this cohort study, we performed a secondary analysis of 2 prospective, multicenter cohort studies of survivors of ARF conducted in the US between January 2019 and August 2022 (NCT03797313 and NCT03738774). We included all 224 survivors of ARF who were negative for COVID-19 and had health expectation data enrolled in either parent study. All sites had institutional review board approval and obtained informed consent. This study is reported following the Strengthening the Reporting of Observational Studies in Epidemiology (STROBE) reporting guideline.

Our primary outcome was *expected health*, a numerical estimate for health 6 months after hospitalization for ARF, reported within 4 weeks of hospital discharge. Expected health was assessed using a validated scale ranging from 0 to 100.^1,3^ Previous work has identified 8 points as a clinically meaningful change on this scale.^1,4^

We estimated the association of having non–COVID-19 ARF with expected health after the COVID-19 pandemic’s first peak using a regression discontinuity design. The first peak was defined as the time between the start of the pandemic (March 11, 2020) and the end of the week of all-time maximal search engine (Google) searches for *COVID19* in the US (March 28, 2020).^5^ Additional exploratory models were fit using June 30, 2020, and November 30, 2021, as crossover dates, representing the end of the pandemic’s first wave and the start of peak searches for *Long COVID*, respectively, as determined by Google Trends.^5^ Each regression discontinuity model used multivariable linear regression, including a before vs after variable, time (days), and an interaction term between time and the before vs after variable.

We adjusted for age, race (self-identified at patient registration and reported in the medical record), sex, formal education (years), median income of home zip code, admitting diagnosis, APACHE II score, presence of acute respiratory distress syndrome, prehospitalization comorbidity burden, hospital length of stay, and type of respiratory support. We included sex and race because they may be associated with health outcomes and perceived recovery. We treated 2-sided *P* values <.05 as statistically significant. Statistical analysis was performed using RStudio version 2022.2.1.461 (RStudio).

## Results

Among 224 participants (median [IQR] age, 53 [43-64] years; 54.9% male; 63.4% White and 28.6% Black or African American) (Table), the median (IQR) expected 6-month health was 90 (75-100) of 100. Enrollment after vs before the pandemic’s first peak was not associated with a change in expected health (4.7 points; 95% CI, −4.0 to 13.3 points; *P* = .29) (Figure). There was similarly no difference in expected health among survivors enrolled on or after vs before June 30, 2020 (7.2 points; 95% CI, −2.5 to 16.9 points) or on or after vs before November 30, 2021 (4.4 points; 95% CI, −7.4 to 16.1 points). There was an interaction between time and enrollment after November 2021, with a change in expectations of −0.08 points per day (95% CI, −0.15 to −0.01 points/d; *P* = .03) on or after November 30, 2021.

**Table.** Characteristics of Study Population

Characteristic	Survivors of ARF, No. (%)^a^		
	On or before March 11, 2020 (n = 107)	After March 28, 2020 (n = 117)	Total (N = 224)
Age, median (IQR), y	53 (42-64)	54 (43-63)	53 (43-64)
Sex			
Male	61 (57.0)	62 (53.0)	123 (54.9)
Female	46 (43.0)	55 (47.0)	101 (45.1)
Race			
Black or African American	23 (21.5)	41 (35.0)	64 (28.6)
White	78 (72.9)	64 (54.7)	142 (63.4)
Other^b^	6 (5.6)	12 (10.3)	18 (8.0)
Ethnicity			
Not Hispanic or Latino	103 (96.3)	111 (94.9)	214 (95.5)
Hispanic or Latino	3 (2.8)	4 (3.4)	7 (3.1)
Highest level of education			
<High school	17 (15.9)	13 (11.1)	30 (13.4)
High school graduate or GED	23 (21.5)	35 (29.9)	58 (25.9)
Some college or 2-y degree	31 (29.0)	23 (19.7)	54 (24.1)
4-y College graduate	17 (15.9)	23 (19.7)	40 (17.9)
>4-y College degree	12 (11.2)	13 (11.1)	25 (11.2)
Unknown or missing	7 (6.5)	10 (8.5)	17 (7.6)
Annual income of home zip code, median (IQR), $	72 206 (52 334 to 87 702)	76 675 (57 835 to 99 221)	73 646 (55 216 to 94 797)
Charlson Comorbidity Index score, median (IQR)	2 (0-3)	1 (0-3)	1 (0-3)
Functional Comorbidity Index score, median (IQR)	2 (1-3)	2 (1-3)	2 (1-3)
Met criteria for ARDS	26 (24.3)	52 (44.4)	78 (34.8)
APACHE II score, median (IQR)	21 (15-26)	22 (15-27)	21 (15-27)
Respiratory support			
Endotracheal tube	84 (78.5)	87 (74.4)	171 (76.3)
HFNC	18 (16.8)	15 (12.8)	33 (14.7)
CPAP or BiPAP	5 (4.7)	5 (4.3)	10 (4.5)
Missing	0	10 (8.5)	10 (4.5)
Length of hospital stay, median (IQR), d	14 (10-22)	14 (10-23)	14 (10-23)
Expected health in 6 mo, median (IQR)	80 (75-95)	90 (80-100)	90 (75-100)

Abbreviations: APACHE II, Acute Physiology and Chronic Health Evaluation; ARDS, acute respiratory distress syndrome; ARF, acute respiratory failure; BiPAP, bilevel positive airway pressure; CPAP, continuous positive airway pressure; GED, General Educational Development test; HFNC, high-flow nasal cannula.

^a^ ARF was defined as requirement of mechanical ventilation (not for mental status only), noninvasive ventilation (CPAP or BiPAP) for 24 or more consecutive hours (not for obstructive sleep apnea or other stable use), or HFNC with a fraction of inspired oxygen of 0.5 or greater and a flow rate of 30 L/min for 24 or more consecutive hours.

^b^ Other includes Asian, American Indian or Alaska Native, Native Hawaiian or Other Pacific Islander, not specified, and unknown. These categories were combined due to small population sizes.

**Figure.**
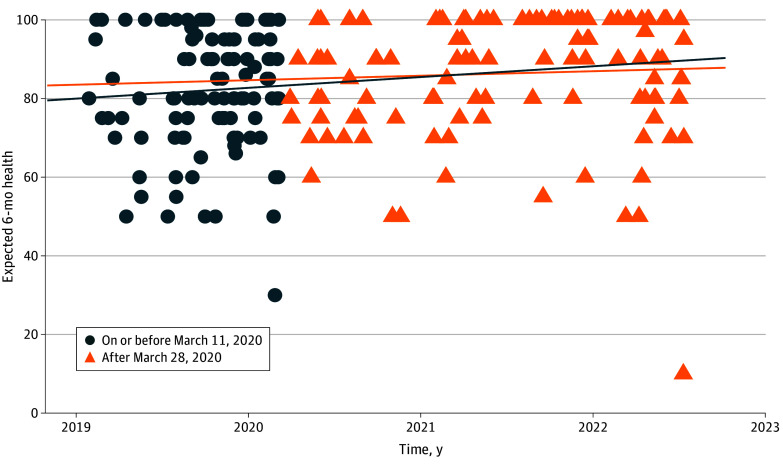
Expected 6-mo Health Before and During the COVID-19 Pandemic This figure displays expected health 6 months after acute respiratory failure estimated by patients discharged from the hospital between January 20, 2019, and July 1, 2022. Separate linear regression lines are displayed for patients asked about their expected health on or before March 11 and after March 28, 2020. Regression lines show the association between time in days and expected 6-month health, adjusted for age, race (reported in the medical record), sex, formal education (years), median income of home zip code, admitting diagnosis, Acute Physiology and Chronic Health Evaluation (APACHE) II score, presence of acute respiratory distress syndrome, prehospitalization comorbidity burden, hospital length of stay, and type of respiratory support.

## Discussion

In this cohort study, we found no clinically meaningful change in expectations about recovery during the COVID-19 pandemic among survivors of non–COVID-19 ARF; expectations were high and similar to previously described expectations in survivors of ARF.^6^ We found a signal that expectations may have begun to lower on or after November 30, 2021.

While limited by the study’s retrospective nature, our findings suggest that despite increased availability of information regarding long-term sequelae of ARF during the COVID-19 pandemic, survivors of non–COVID-19 ARF did not integrate this information as relevant to their health. Historically, post–intensive care unit (ICU) disability has been poorly appreciated, and our results suggest that this remains the case. Normalizing appropriate expectations about recovery will require creative partnership with patients in the ICU to understand how to amplify realistic and relevant information about recovery.

## Notes

Supplement 1. **Nonauthor Collaborators.** Addressing Post-Intensive Care Syndrome (APICS) Study Team members

Supplement 2.
Data Sharing Statement
