# Supplementary material for: The COVID-19 Pandemic and Patient Expectations About Recovery From Acute Respiratory Failure
Source: JAMA Netw Open. 2024 Nov 8;7(11):e2444318. doi: 10.1001/jamanetworkopen.2024.44318 (PMC11549653; doi:10.1001/jamanetworkopen.2024.44318)
Supplement: Supplement 1. — Nonauthor Collaborators. Addressing Post-Intensive Care Syndrome (APICS) Study Team members [file jamanetwopen-e2444318-s001.pdf]

*Group Name(s): Addressing Post-Intensive Care Syndrome (APICS) Study Team							
*First Name and Middle Initial(s)	*Last Name	*Suffix (eg, Jr, III)	Academic Degrees	Institution	Location (city, state/province, country)	Role or Contribution, eg, chair, principal investigator	Group (if more than 1 Group listed in the byline) and/or Subgroup (eg, Steering Committee)
Krystal	Capers	N/A		Beth Israel Deaconess Medical Cente	Boston, MA, USA		
Julia	Crane	N/A		Beth Israel Deaconess Medical Cente	Boston, MA, USA		
Benjamin	Hoenig	N/A		Beth Israel Deaconess Medical Cente	Boston, MA, USA		
Maria	Karamourtopoulos	N/A		Beth Israel Deaconess Medical Cente	Boston, MA, USA		
Julia	Larson	N/A		Beth Israel Deaconess Medical Cente	Boston, MA, USA		
Andre	De Souza Licht	N/A		Beth Israel Deaconess Medical Cente	Boston, MA, USA		
Lauryn	Tsai	N/A		Beth Israel Deaconess Medical Cente	Boston, MA, USA		
Narges	Akhlaghi	N/A		Johns Hopkins University	Baltimore, MD, USA		
Omar	Almaadawy	N/A		Johns Hopkins University	Baltimore, MD, USA		
Toan	Bui	N/A		Johns Hopkins University	Baltimore, MD, USA		
Shihua	Chen	N/A		Johns Hopkins University	Baltimore, MD, USA		
Sai Phani Sree	Cherukuri	N/A		Johns Hopkins University	Baltimore, MD, USA		
Nicole	Illesca	N/A		Johns Hopkins University	Baltimore, MD, USA		
Ehizele	Iyayi	N/A		Johns Hopkins University	Baltimore, MD, USA		
Preethi	Kadiri	N/A		Johns Hopkins University	Baltimore, MD, USA		
Teja	Kalva	N/A		Johns Hopkins University	Baltimore, MD, USA		
Mounica	Koneru	N/A		Johns Hopkins University	Baltimore, MD, USA		
Mazin	Ali Mahmoud	N/A		Johns Hopkins University	Baltimore, MD, USA		
Albahi	Malik	N/A		Johns Hopkins University	Baltimore, MD, USA		
Roozbeh	Nikooie	N/A		Johns Hopkins University	Baltimore, MD, USA		
Darin	Roberts	N/A		Johns Hopkins University	Baltimore, MD, USA		
Bhavna	Seth	N/A		Johns Hopkins University	Baltimore, MD, USA		
Sriharsha	Singu	N/A		Johns Hopkins University	Baltimore, MD, USA		
Parvaneh	Vaziri	N/A		Johns Hopkins University	Baltimore, MD, USA		
Darrin	Applegate	N/A		Intermountain Medical Center	Murray, UT, USA		
Valerie	Aston	N/A		Intermountain Medical Center	Murray, UT, USA		
Katie	Brown	N/A		Intermountain Medical Center	Murray, UT, USA		
Melissa	Fergus	N/A		Intermountain Medical Center	Murray, UT, USA		
Ellie	Hirshberg	N/A		Intermountain Medical Center	Murray, UT, USA		
Naresh	Kumar	N/A		Intermountain Medical Center	Murray, UT, USA		
Jenna	Lumpkin	N/A		Intermountain Medical Center	Murray, UT, USA		

***First Name and Middle Initial(s)**	***Last Name**	***Suffix (eg, Jr, III)**	Academic Degrees	Institution	Location (city, state/province, country)	Role or Contribution, eg, chair, principal investigator	Group (if more than 1 Group listed in the byline) and/or Subgroup (eg, Steering Committee)
Erna	Serezlic	N/A		Intermountain Medical Center	Murray, UT, USA		
Rilee	Smith	N/A		Intermountain Medical Center	Murray, UT, USA		
Craig	High	N/A		Salt Lake City Veterans	Salt Lake City, UT, USA		
Emily	Beck	N/A		Salt Lake City Veterans	Salt Lake City, UT, USA		
Rebecca	Abel	N/A		Vanderbilt University Medical Center	Nashville, TN, USA		
Liza	Frawley	N/A		Vanderbilt University Medical Center	Nashville, TN, USA		
Margaret	Hays	N/A		Vanderbilt University Medical Center	Nashville, TN, USA		
Susan	Mogan (in memoriam)	N/A		Vanderbilt University Medical Center	Nashville, TN, USA		
