# Supplementary material for: The COVID-19 Pandemic and Patient Expectations About Recovery From Acute Respiratory Failure
Source: JAMA Netw Open. 2024 Nov 8;7(11):e2444318. doi: 10.1001/jamanetworkopen.2024.44318 (PMC11549653; doi:10.1001/jamanetworkopen.2024.44318)
Supplement: Supplement 2. — Data Sharing Statement [file jamanetwopen-e2444318-s002.pdf]

Data Sharing Statement

Bouhassira. The COVID-19 Pandemic and Patient Expectations About Recovery From Acute Respiratory Failure. *JAMA Netw Open*. Published online November 8, 2024. doi:10.1001/jamanetworkopen.2024.44318

Data
Data available: No
